# Natural Agents as Novel Potential Source of Proteasome Inhibitors with Anti-Tumor Activity: Focus on Multiple Myeloma

**DOI:** 10.3390/molecules28031438

**Published:** 2023-02-02

**Authors:** Francesca Alessandra Ambrosio, Giosuè Costa, Maria Eugenia Gallo Cantafio, Roberta Torcasio, Francesco Trapasso, Stefano Alcaro, Giuseppe Viglietto, Nicola Amodio

**Affiliations:** 1 Department of Experimental and Clinical Medicine, Campus “S. Venuta”, University “Magna Græcia” of Catanzaro, Viale Europa, 88100 Catanzaro, Italy; 2 Department of Health Sciences, University “Magna Græcia” of Catanzaro, Campus “S. Venuta”, Viale Europa, 88100 Catanzaro, Italy; 3 Net4Science Academic Spin-Off, University “Magna Græcia” of Catanzaro, Campus “S. Venuta”, Viale Europa, 88100 Catanzaro, Italy; 4 Department of Biology, Ecology and Earth Sciences (Di.B.E.S.T.), University of Calabria, 87036 Rende, Italy; 5 Associazione CRISEA—Centro di Ricerca e Servizi Avanzati per l’Innovazione Rurale, Loc. Condoleo, 88055 Belcastro, Italy

**Keywords:** proteasome, natural compounds, proteasome inhibitors, multiple myeloma

## Abstract

Multiple myeloma (MM) is an aggressive and incurable disease for most patients, characterized by periods of treatment, remission and relapse. The introduction of new classes of drugs, such as proteasome inhibitors (PIs), has improved survival outcomes in these patient populations. The proteasome is the core of the ubiquitin–proteasome system (UPS), a complex and conserved pathway involved in the control of multiple cellular processes, including cell cycle control, transcription, DNA damage repair, protein quality control and antigen presentation. To date, PIs represent the gold standard for the treatment of MM. Bortezomib was the first PI approved by the FDA, followed by next generation of PIs, namely carfilzomib and ixazomib. Natural agents play an important role in anti-tumor drug discovery, and many of them have recently been reported to inhibit the proteasome, thus representing a new potential source of anti-MM drugs. Based on the pivotal biological role of the proteasome and on PIs’ significance in the management of MM, in this review we aim to briefly summarize recent evidence on natural compounds capable of inhibiting the proteasome, thus triggering anti-MM activity.

## 1. Introduction

Multiple myeloma (MM) is the second most common hematologic malignancy worldwide, characterized by the proliferation of terminally differentiated antibody-producing plasma cells (PCs) [1,2]. Although MM has intrinsic genetic heterogeneity [3], a common feature of malignant clones is the production of elevated amounts of immunoglobulins [1], ultimately leading to organ dysfunctions such as hypercalcemia, renal insufficiency, anemia and bone disease (overall referred to as CRAB criteria) [4,5]. The bone marrow microenvironment (BMM) has been shown to play a significant role in MM pathogenesis triggering PC survival, proliferation and drug resistance [1,2]; moreover, genetic complexity, defined by chromothripsis and hyperdiploidy, along with copy number variations and single-nucleotide polymorphisms, account for the early evolution from asymptomatic stages (e.g., monoclonal gammopathy of undetermined significance (MGUS) and smoldering multiple myeloma (SMM)) to overt disease [6]. Additional alterations, including aberrant DNA or histone methylation [7] and microRNA (miRNA) dysregulation [8,9], may contribute to disease progression.

The ubiquitin–proteasome system (UPS) is crucial homeostatic machinery for protein degradation; it constantly regulates protein turnover, thus affecting various cellular functions, spanning from cell cycle regulation to survival, apoptosis, metabolism and protein quality control [10]. Since PCs secrete high amounts of immunoglobulins, they are strictly dependent on the UPS machinery and are highly reliant on the deregulation of protein degradation. Moreover, due to the constitutive activation of the NF-κB signaling pathway, malignant PCs are more sensitive to PI than healthy ones [11]. In fact, the anti-MM activity of proteasome inhibitors (PIs) was originally ascribed to the inhibition of oncogenic NF-κB through the blockage of the degradation of its negative regulator IκBα [12]. However, additional processes whose targeting contributes to the anti-tumor effects of PIs have subsequently been identified, including the reversal of cell cycle aberrations, apoptosis induction, endoplasmic reticulum stress, angiogenesis and DNA repair, as well as the reactivation of hypermethylated tumor-suppressor genes [12,13,14,15].

The exquisite sensitivity of MM cells to PIs, along with the design of successful clinical protocols, have led to their approval for the treatment of MM patients, with three compounds currently being used in clinics [16]. PIs are true milestones for the treatment of MM and other hematologic malignancies (e.g., mantle cell lymphoma), and are currently being investigated for other diseases. The first approved PI was bortezomib, a slowly reversible inhibitor of the β5 catalytic proteasomal subunit, followed by carfilzomib, an irreversible inhibitor of the β5 site, and by the first oral PI, ixazomib [2].

Natural products have always played a key role in drug discovery, especially for cancer and infectious diseases. In oncology, several drugs derived from Nature have been approved, and are widely used in clinics, including paclitaxel, romidepsin, vincristine and vinblastine [17,18,19,20].

Importantly, marine-derived natural compounds isolated from sponges, mollusks, cyanobacteria, corals and tunicates have been clinically approved for MM by the US and Australian FDA as well as the European Medicines Agency (EMA), such as belantamab mafodotin and plitidepsin, while others are under clinical trial or extensive preclinical assessment [21].

Additionally, naturally derived compounds with PI activity have been isolated from various sources, and are currently emerging as potential anti-cancer drugs in a wide variety of cancers, including MM [22].

In the present review, we aim at summarizing key aspects of proteasome structure and function, pointing to recent preclinical research on emerging natural compounds endowed with PI activity. Overall, findings in the literature underscore the promising role of these molecules as drug candidates/lead compounds for the management of MM and likely other proteasome-addicted diseases.

## 2. Proteasome Structure and Function

By ruling protein degradation, the UPS represents a fundamental intracellular system whose components act in a highly coordinated manner through different steps, such as the polyubiquitylation, deubiquitylation and degradation of the target protein [16,23]. Thanks to the coordinated activities of this system, all the damaged or misfolded proteins, or the proteins which are no longer needed, are correctly destroyed [16]. The proteasome is the central core of this system. The eukaryotic 26S proteasome is a large (1500–2000 kDa) multi-subunit complex that degrades most cellular proteins under physiologic conditions. It is a multicatalytic proteinase complex able to bind, deubiquitylate and unfold its substrates prior to completing their degradation. The proteasome is predominately located in the cytosol and nucleus, and its functions are not limited to the maintenance of proteostasis—it is involved in a wide array of biological processes, including cell differentiation and proliferation, DNA repair and apoptosis, regulation of gene expression and response to stress [24,25]. Alternative forms of the proteasome, such as the immunoproteasome and thymoproteasome, are involved in routine proteolytic functions, antigen processing and T-cell selection [16].

The 26S proteasome is a dynamic ATP-dependent macromolecular machine, comprising two subcomplexes: the 19S regulatory particle (RP) and the 20S core particle (CP).

**Figure 1:**
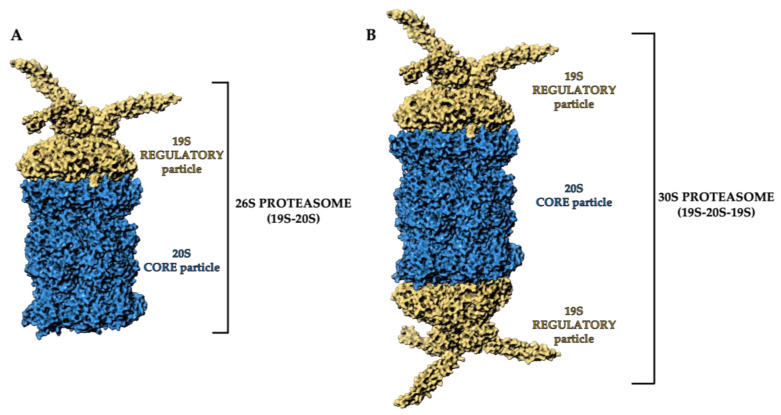
Three-dimensional representation of (**A**) 26S proteasome and (**B**) 30S proteasome structure. The 19S regulatory particles and the 20S core particle are shown as yellow and blue areas, respectively. Atomic coordinates were obtained from PDB model 5MP9 [29]; the figure was built by means of Maestro graphical interface [30].

Either one or two 19S RPs can attach to a single 20S core particle to form the 26S proteasome (19S-20S) (Figure 1A) or the 30S proteasome (19S-20S-19S), respectively. However, both conformations in the literature are referred to as “26S” (Figure 1B) [26,27,28].

The core of the RP is formed by a heterohexameric ATPase associated with various cellular activities (AAA + ATPase), which is the driver of the large-scale conformational dynamics of the RP. The core of the RP prepares substrates for degradation in coordination with at least three ubiquitin receptors (26S proteasome non-ATPase regulatory subunit 1, Rpn1, Rpn10 and Rpn13, and a deubiquitylating subunit, Rpn11). The other subunits mainly have structural roles, such as holding the CP and RP together [29].

The proteasome regulatory subunits bind to each end of the 20S proteasome (Figure 1) and mediate deubiquitylation. These accessory subunits unfold substrates and feed them into the 20S catalytic complex, whilst removing the attached ubiquitin molecules. Once the target proteins have been deubiquitylated, they undergo degradation via the 20S proteasome particle [31], which removes misfolded and damaged proteins, and digests foreign proteins as part of the adaptive immune system [32].

**Figure 2:**
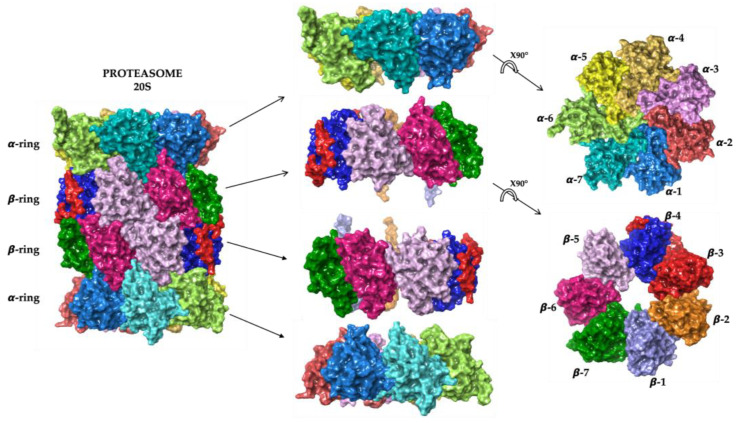
Three-dimensional representation of 20S proteasome structure. Atomic coordinates were obtained from PDB model 5LF7 [33]. The figure was built by means of Maestro graphical interface [30].

The eukaryotic 20S core particle is the catalytic core of this complex, constituted by a cylindrical structure consisting of four heptameric rings containing α and β subunits (Figure 2).

Seven α subunits form the two outer rings and serve as the gates through which proteasome substrates enter, meanwhile the seven β subunits form the inner rings.

The passage through the gates formed by the N-termini of the α subunits is the rate-limiting step, and prevents unregulated protein degradation [34].

The αN-terminal tails are highly conserved, and contain a tyrosine-aspartate-arginine (YDR) motif that forms salt bridges with neighboring tails that obstruct the 13 Å entry pore.

The β subunits contain the N-terminal threonine that provides the nucleophile that attacks the carbonyl group of the peptide bond in the target proteins.

**Figure 3:**
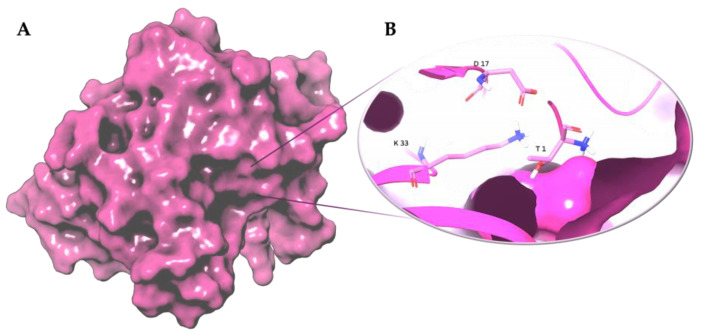
Three-dimensional representation of (**A**) proteasome chymotrypsin-like site (β5); (**B**) Thr-Lys-Asp catalytic triad of the chymotrypsin-like site (β5). The β5 subunit is represented as magenta surface and the conserved residues are shown as magenta carbon sticks. Atomic coordinates were obtained from PDB model 5LF7 [33]; the figure was built by means of Maestro graphical interface [30].

The Thr residue is part of a conserved Thr-Lys-Asp catalytic triad, which functions similarly in both processes [25] (Figure 3). Other important residues for the active site are glycine 129 (Gly129), aspartate 167 (Asp167) and tyrosine 169 (Tyr169).

The Thr1, Asp17 and Lys33 are the most important residues for the proteolytic mechanism meanwhile the other residues are important for the active site integrity [35].

The proteolytically active β subunits, three on each β ring, exhibit different substrate preferences. The proteasome is associated to at least three distinct proteolytic activities, based upon preference to cleave a peptide bond after a particular amino acid residue: the postglutamyl peptidyl hydrolytic-like, the tryptic like and the chymotryptic-like. These proteolytic activities of the proteasome are carried out by the β1, β2 and β5 subunits, respectively [36]. The chymotrypsin-like (β5) activity cleaves after hydrophobic residues, the trypsin-like (β2) sites preferentially cleave after hydrophobic and basic residues, while the caspase-like site (β1) cleaves after the acidic residues [36].

The multiple catalytic sites with varying specificities advantageously allow for the rapid and processive degradation of cellular proteins. As a result, the proteasome is not simply a complex of independent proteases, but a unique multicatalytic enzyme properly functioning when fully intact.

### Approved and Investigational PIs for MM Treatment

**Figure 4:**
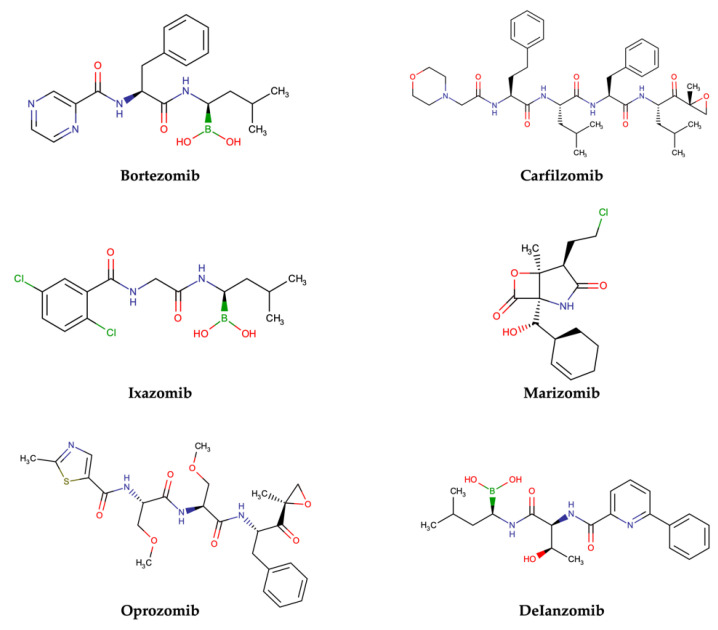
Two-dimensional structures of approved and investigational PIs.

PIs are classified according to their chemical structure and their mechanism of action. Covalent inhibitors are generally electrophilic and react with the catalytic gamma-hydroxyl of Thr1 in the active sites to reversibly or irreversibly inhibit the proteasome depending on the strength of the chemical bond. Based on their chemical structure, PIs can be classified in the following classes: peptide aldehydes, peptide boronates, epoxomicin and epoxyketones, lactacystin, β-lactone and vinyl sulfones. Similar to most protease inhibitors, several PIs are short peptides designed to fit into the substrate binding site of the catalytic subunit. Although the proteasome has three types of catalytic sites, full inhibition of all of them is not required to significantly affect protein degradation. In fact, while β5 inhibition leads to significantly reduced protein breakdown, specific β1 or β2 inhibition does not have any significant effect. Consequently, most PIs act by targeting the β5 site, although they can often have some lesser activity against β1 and/or β2 [37]. To date, three FDA-approved proteasome inhibitors, namely bortezomib (Velcade^®^), carfilzomib (Kyprolis^®^) and ixazomib (Ninlaro^®^), are in clinical use, and several drugs are under development (Figure 4).

**Bortezomib**

Bortezomib (Velcade^®^) (Figure 4), the first reversible PI approved in 2003 by the US Food and Drug Administration (FDA), rapidly became a mainstay in the treatment of relapsed or refractory (and, subsequently, of previously untreated) MM patients [38]. Chemically, bortezomib ([3-methyl-1-(3-phenyl-2- pyrazin-2-ylcarbonylamino-propanoyl) amino-butyl] boronic acid) is a dipeptide boronic acid derivative which contains pyrazinoic acid, phenylalanine and leucine with boronic acid in its structure [39].

Bortezomib targets the chymotrypsin- and caspase-like active sites, with minimal effect on trypsin-like activity (Table 1) [40]. Its usage has been expanded for newly diagnosed MM patients as well as for the treatment of mantle cell lymphoma.

**Carfilzomib**

The tetrapeptide epoxyketone carfilzomib (Kyprolis^®^) (Figure 4) is the first irreversible PI approved by the FDA for the treatment of relapsed and refractory MM [39].

Carfilzomib differs from bortezomib in structure, activity and the irreversibility of its binding mode, showing potent and selective inhibition of chymotrypsin-like activity with lower affinity for the trypsin- and caspase-like proteases (Table 1). Carfilzomib is active in vitro against bortezomib-resistant MM cell lines, as well as in vivo in patients with bortezomib-resistant MM.

**Ixazomib**

Ixazomib (Ninlaro^®^) (Figure 4) is an orally bioavailable, reversible PI developed by Takeda Oncology, approved by the FDA in 2015. It is made up of an alanine leucine dipeptide core, and it acts as a reversible inhibitor of the chymotrypsin-like β5 subunit of the 20S proteasome [41].

**Marizomib**

Marizomib (salinosporamide A) is a marine-derived extract from Actinomycete strains, identified as a member of the Micromonospora family at Scripps Institution of Oceanography (SIO) in the late 1980s. With the help of DNA sequencing (16S rRNA gene sequencing), these strains were found to be distinctive from all Micromonospora species, and were named as a new genus, *Salinospora* [42]. Chemically, marizomib is a bicyclic β-lactone γ-lactam acting as a broad-spectrum PI able to irreversibly bind the three major catalytic β5, β1 and β2 sites (Figure 4–Table 1), demonstrating efficacy in relapsed and/or refractory MM patients becoming resistant to bortezomib, carfilzomib and ixazomib regimens [43,44].

**Oprozomib**

Oprozomib (ONX 0912) (Figure 4) is an oral analog of carfilzomib, belonging to the epoxyketone PIs. It inhibits the β5 subunit like carfilzomib, demonstrating an equivalent anti-tumor activity to carfilzomib in in vitro and in vivo models.

**Delanzomib**

Delanzomib (CEP-18 770) (Figure 4) is related to bortezomib, with a peptide-like backbone and a boronate warhead. It is an oral and reversible β5 subunit proteasome inhibitor [43].

**Table 1:** Name, pharmacokinetics and pharmacodynamics characteristics of proteasome inhibitors.

Compound	Pharmacophoric Moiety	Binding Kinetics	Proteasome Subunit
Bortezomib	Boronate	Reversible	β5 > β1
Carfilzomib	Epoxyketone	Irreversible	β5
Ixazomib	Boronate	Reversible	β5 > β1
Marizomib	β-lactone	Irreversible	β5 > β1 > β2
Oprozomib	Epoxyketone	Irreversible	β5
Delanzomib	Boronate	Reversible	β5 > β1

## 3. Natural PIs in MM

Although PIs are overall clinically safe, peculiar adverse profiles (i.e., peripheral neuropathy and cardiotoxicity) frequently arise during PI-based regimens. To overcome this drawback, several strategies, such as the use of combinatorial treatments, have been designed with the aim of lowering the dose of PIs, therefore minimizing their side-effects [12]. As a further approach, naturally derived molecules with PI activity have been discovered and characterized as alternative PIs. The relevance of natural compounds is underscored by the fact that, in the last four decades, about a quarter of all approved drugs are of natural origin (not including biologicals), while another quarter are inspired by Nature [17,18]. This is even more the case for anticancer drugs, for which the 25% of the approved compounds are of natural origin or Nature-derived [45]. Given the relevance of the proteasome as therapeutic target in MM, the search for natural PIs has significantly intensified in the last several decades [21].

In the following sections, we will examine the natural agents which have been reported to be endowed with PI activity in preclinical models of MM (see Table 2 for a summary).

### 3.1. Tea Polyphenols

Black tea is a polyphenol-rich aqueous infusion of the dried leaves of the plant *Camellia sinensis*. Tea consumption has been reported to have several health benefits, including decreased incidence of cancer [46]. Many efforts have been made to unravel the molecular mechanisms underpinning the cancer-preventive properties of tea, which have been associated to its content in antioxidants, likely protecting against oxidative stress or mutagens [47]. Theaflavins (TFs) and thearubigins are the major constituents found in black tea, accounting for its unique taste and bright red-orange color. Thearubigins have higher molecular weight and are poorly characterized compared to TFs. The flavins have a benzotropolone ring structure and are a mixture of theaflavin (TF-1), theaflavin-3-gallate (TF-2a), theaflavin-3′-gallate (TF-2b) and theaflavin-3,3′-digallate (TF-3).

TF-1 is considered the most bioactive and protective agent against carcinogens. Black tea polyphenols have been reported to inhibit proliferation and induce apoptosis in a variety of tumor models by promoting the tumor suppressor p53 and down-regulating nuclear factor kappa B (NF-κB) and mitogen-activated protein kinase (MAPK) pathways [48,49,50]. Both polyphenon 60 (green tea extract) and T5550 (black tea extract) were shown to significantly inhibit the CT-like activity of purified 20S proteasome at concentrations of 10 and 20 μg/mL, respectively. In vitro experiments using MM cell lines showed that the black tea extract, namely T5550, but not the green tea extract, polyphenon 60, potently dampened the CT-like proteasome activity. Through computational docking studies, Mujtaba et al. identified TF-1 as susceptible to nucleophilic attack by the N-terminal threonine of the subunit β5 of proteasome, leading to a dose-dependent block of the CT-like and trypsin-like activities of the proteasome [51].

Unexpectedly, some studies have highlighted that various green tea constituents, in particular (-)-epigallocatechin gallate (EGCG) and other polyphenols with 1,2-benzenediol moieties, effectively prevented bortezomib-induced tumor cell death. In this regard, Golden et al. showed severe antagonism during combined treatment with EGCG and bortezomib, as indicated by findings that EGCG or green tea extract effectively blocked bortezomib-induced proteasome inhibition and ER stress, and subsequent tumor cell death in vitro and in vivo. The severe antagonistic effect of EGCG appeared related to the boronic acid moiety of the bortezomib, since in the study the authors report that among the PIs, the boronic acid-containing ones were similarly inactivated by EGCG, whereas none of those lacking this functional group were affected. Indeed, the primary mechanism of EGCG’s antagonism resides in the chemical structure of the target molecule (i.e., the boronic acid moiety). Since blockade of bortezomib’s anti-MM activity was obtained at concentrations of EGCG that are commonly achieved in humans, these findings recommend caution when green tea products are administered to patients undergoing bortezomib-based regimens. Overall, further studies on tea polyphenols are required to draw conclusions regarding the protective/antagonistic effect of black and green tea polyphenols towards PI-based therapy [52].

### 3.2. Hib-Ester and Hib-Carbaldehyde

*Hibiscus sabdariffa* (*H. sabdariffa*) is an annual herbaceous subshrub belonging to the Malvaceae family, also known as Roselle, Red Sorrel or Karkadè, commonly distributed in tropical and subtropical regions but originally native to India and Saudi Arabia. *H. sabdariffa* is cultivated in regions such as Sudan, Egypt, Nigeria, Mexico, Saudi Arabia, Taiwan, West Indies, Central America and European countries [23,53]. *H. sabdariffa* calyces are commonly used for the preparation of tea and infusions. The red drink is widely consumed directly, or used for the preparation of juice, jam, pudding and other foods.

The main components of *H. sabdariffa* extracts are phenols, polyphenols, anthocyanins and organic acids, such as citric, tartaric, malic and ascorbic acids.

*H. sabdariffa* was initially shown to elicit antiproliferative effects against breast, ovarian and cervical cancer cells, as well as leukemia cells [23,54]. In 2021, by a microwave-assisted solvent extraction (MASE) procedure exploiting 80% ethanol as the extracting solvent, Malacrida et al. obtained the two active compounds of H. sabdariffa, namely Hib-ester and Hib-carbaldehyde.

Hib-ester and Hib-carbaldehyde exerted in vitro anti-tumor activity against MM RPMI 8226 and U266.B1 cell lines, with IC_50_ values at 24 h of 0.45 and 0.21 mg/mL, respectively; moreover, both compounds were effective at non-neurotoxic µg/mL concentrations. The authors investigated their possible biological sequelae, showing Hib-ester and Hib-carbaldehyde as capable of triggering apoptosis while inhibiting autophagy. In addition, molecular modelling studies were carried out, outlining a good interaction of both compounds with the β5-chymotrypsin active site. Both the enriched fraction of the extract (HsEF) and its metabolites were able to significantly impair the proteasome activity, with the HsEF being more effective, likely because of the anthocyanins content of the *H. sabdariffa* ethanolic extract, previously shown to be endowed with PI activity [23].

### 3.3. Celastrol

Celastrol is a quinone methide triterpene isolated from the Chinese medicinal plant *Tripterygium wilfordii*, also known as “Thunder of God Vine” and Lei Gong Teng. In the past, the “Thunder of God Vine” has been used by certain populations for the treatment of autoimmune diseases, chronic inflammation and neurodegenerative diseases such as arthritis, lupus erythematosus, lateral sclerosis and Alzheimer’s disease. More recently, celastrol attracted attention due to its potent anticancer activities, as it suppressed the proliferation of different human cancer cell lines and inhibited the growth of prostate, breast and lung cancer xenograft models. Lastly, in 2019, celastrol was shown to exert inhibitory activity on the proliferation of MM cells [55]. In detail, Zhong Y. et al. reported that celastrol could inhibit the proliferation of MM cells through the inhibition of the caspase-like (β1), trypsin-like (β2) and chymotrypsin-like (β5) proteasome activities of purified human 20S proteasomes, as corroborated by in vitro assays, with half-maximal inhibitory concentration (IC50) values of 7.1, 6.3 and 9.3 μmol/L, respectively [55].

In the same study, it was reported that celastrol, by inhibiting proteasome activity, triggers apoptosis and cell-cycle blockade of tumor cells; in the same study, the authors reported interesting in vivo studies showing that tumor growth in celastrol-treated mice was significantly affected compared with that of the controls. At the end of the celastrol treatment of mice xenografted with MM.1S and RPMI8226 MM cells, the average tumor size in the respective control groups was 1296.65 ± 568.02 mm^3^ and 789.10 ± 117.51 mm^3^, respectively, whereas the tumor size in the celastrol-treated groups was 1831.88 ± 949.24 mm^3^ and 1522.15 ± 569.04 mm^3^, respectively. The average tumor weight of the respective control groups was 1046.0 ± 570.0 mg and 918.4 ± 289.3 mg, whereas that of the celastrol-treated groups was 640.9 ± 415.4 mg and 457.0 ± 91.3 mg. Regarding the body weight loss, treated animals showed minimal body weight loss at the end of the celastrol treatment [55].

### 3.4. Syrbactins

Some strains of *Pseudomonas syringae pv. Syringae* (Pss), a pathogen of many plant species causing brown spot disease on bean, secrete syringolin (SylA), a peptide derivative synthesized by a mixed non-ribosomal peptide synthetase (NRPS)/polyketide synthetase (PKS) [56]. Transcriptome profiling showed that the exogenous application of SylA on wheat and *Arabidopsis thaliana* significantly perturbed gene expression, with transcripts encoding all proteasome subunits and many heat-shock proteins accumulating in *Arabidopsis* leaves. SylA irreversibly reacted with the N-terminal Thr of the active site in the β5 pocket by a 1,4-addition of the hydroxyl group of the Thr to the α,β-unsaturated carboxamide moiety of SylA. Notably, SylA was found to inhibit all proteasome activities in vitro, with the chymotrypsin-like activity being the most sensitive. Accordingly, the SylA treatment of neuroblastoma cells triggered a time-dependent increase in the protein levels of the tumor suppressor p53, a known target of the proteasome [57]. Thereafter, the synthesis, computational affinity assessment and preclinical evaluation of TIR-199, a natural-product-derived syrbactin structural Syl-analog, was performed by the same authors. Molecular modeling and simulation suggested that TIR-199 covalently binds each of the three catalytic subunits (β1, β2 and β5). In vitro and cell-culture-based proteasome activity assays confirmed that TIR-199 dose-dependently inhibited the proteasome and induced tumor cell death in MM, neuroblastoma cells as well as other cancer types in the NCI-60 cell panel. In vivo studies in mice revealed a maximum tolerated dose of TIR-199 of 25 mg/kg, and the anti-tumor activity of TIR-199 was confirmed in hollow fiber assays in mice. Importantly, adverse drug reaction screens in a kidney panel did not highlight any significant off-target effects [58].

Subsequently, TIR-199 was found to selectively inhibit to varying degrees the three sub-catalytic proteasomal activities, C-L/β1, T-L/β2 and CT-L/β5, in three MM cell lines, resulting even more effective than bortezomib, carfilzomib and ixazomib in killing bortezomib-resistant MM and mantle cell lymphoma cell lines, as suggested by a lower resistance index [59]. It is noteworthy that mice treated with TIR-199 showed reduced MM burden and improved trabecular bone numbers and smaller gaps between the bones, suggesting improved bone health and potential anti-bone disease activity [60].

### 3.5. Tyropeptins

Tyropeptins are natural compounds extracted from microbial metabolites, isolated in the year 2000 from the culture broth of the Actinomycete strain MK993-dF2, forming well-branched vegetative mycelia and aerial hyphae that bore spirals, generally growing at 10–37 °C, and utilizing sugars such as D-glucose, D-mannitol and D-xylose [61]. Chemically, tyropeptins are peptide aldehyde inhibitors containing an aldehyde moiety at the C-terminal, with strong inhibitor activity on the proteasome which relies on a hemiacetal formation with the hydroxyl of the threonine active site [62]. These compounds were found to inhibit the chymotrypsin-like and trypsin-like of the human-erythrocyte-derived 20S proteasome, while sparing caspase activity. Tyropeptin A penetrates cell membranes inhibiting the chymotrypsin-like and trypsin-like activity of the 20S proteasome, with IC50 values of 0.10 µg/mL and 1.5 µg/mL respectively [61,63]. In detail, the inhibition occurred on the substrate binding site formed by the association of the β5 subunit with β6, leading to the accumulation of endogenous ubiquitinated proteins. To improve the PI inhibitory activity, Momose et al. synthesized several tyropeptin-derived boronic acid derivatives, with replacement of the isopropyl with a cyclohexyl group, eliciting an inhibitory effect four times stronger than that of tyropeptin A [64]. Two of these compounds, namely AS-06 and AS-29, induced the accumulation of ubiquitinated proteins in human MM cells, suppressing the degradation of IKB-α and the nuclear translocation of p65 in MM cells; they also triggered apoptosis through caspase-8 and caspase-9 activation and cytochrome c release from mitochondria, while decreasing the caspase inhibitors c-IAP-1 and XIAP. Importantly, AS-29 also elicited anti-cancer activity in in vivo models, as demonstrated by the observation that its intravenous administration (4.0 mg/kg) significantly reduced the growth of sub-cutaneous MM xenografts without exerting any apparent toxic effect [61,63,65].

### 3.6. Pristimerin

Pristimerin is a naturally occurring quinonemethide triterpenoid, isolated from root cortex of plants belonging to *Celastraceae* or *Hippocrateaceae* species, found in Myatenus, Crossopetalum, Celastrus, Cheiloclinium and Mortonia plants distributed in Latin America, Northern Mexico and the Southern United States [66,67]. Triterpenoids are biosynthesized by plants through the cyclization of squalene, leading to different organic chemicals with a similar basic backbone consisting of six linked isoprene units [67]. Triterpenoids such as betulinic acid and the ginsenosides possess anticancer activities in vitro and in vivo that appear to be mediated, at least in part, by their ability to block TNF-induced NF-kB activation via inhibition of IKKα, IKKβ or IkBα.

Pristimerin is a natural biologically active molecule which has long been used as a traditional medicine for the cure of different diseases in Latin America [68]. Various pharmacological effects of primisterin, including anti-inflammatory, cytotoxic, antioxidant, antimalarial and insecticidal properties, have been reported [67].

Many studies have also reported the clinical potential of pristimerin as an anti-proliferative agent, at a concentration range (IC_50_) of 0.2–4 µM, against different human solid and hematologic malignancies, including leukemia and MM [69,70,71,72,73,74]. In the latter case, pristimerin isolated from Celastrus and Maytenus species displayed significant anti-MM activity, with an IC_50_ < 100 nM, leading to selective apoptosis in vitro and in vivo in a plasmacytoma model, which was associated with the inhibition of both NF-kB pathway and cyclin D expression, and to proteasome blockade. Accordingly, the direct effect of pristimerin on proteasome enzymatic function was demonstrated by the rapid and potent inhibition of the chymotrypsin active site at low concentrations (less then 100 nM), both in a cell-free system exploiting purified 20S proteasome, and in cell-based proteasome assays.

### 3.7. Ursolic Acid

Pentacyclic triterpenes and their derivatives have highly diverse structures that are widely present in the plant kingdom, and are especially abundant in apple peel, displaying various anti-tumor, anti-inflammatory, anti-viral and antioxidant activities [75,76]. Within the triterpenes, ursolic acid (UA) exhibits significant anti-inflammatory and anti-cancer activities, affecting Nrf2, NF-κB, STAT3 and AKT signaling pathways [77]. In MM cells, UA treatment led to the inhibition of both inducible and constitutive STAT3 activities, mainly through the induction of the mRNA transcript of SHP-1, a non-transmembrane protein tyrosine phosphatase abundantly expressed in hematopoietic cells [78,79]. Moreover, UA induced the apoptosis of MM cells by upregulating the expression of Bax and Bak proteins [80]. USPs deubiquitinases such as USP1, USP2, USP7, USP9x and USP14 have been implicated in cancer onset and progression [81]. Among them, USP7 has been thoroughly investigated in cancer pathophysiology. USP7 inhibition was found to target MDM2, thus upregulating p53, leading to cell cycle arrest and apoptosis of cancer cells [82]. Moreover, many oncosuppressive proteins were shown to be USP7 substrates, including PTEN [83]. Elevated expression of USP7 has been observed in MM, and inhibition of USP7 can antagonize proliferation and induce cell death [84], thus representing a potential anti-tumor strategy [85]. In 2018, Jing et al. reported that UA interacts with USP7, and through molecular docking analysis showed that UA might occupy the ubiquitin-binding pocket of USP7, with the 17-carboxyl group and 3-hydroxyl group playing a vital role in the USP7-UA interaction. SAR analysis performed by Jing et al. suggested the number and position of triterpenes’ hydroxyl groups as relevant for the potency of the molecules. Consistently, ester conversion of the 17-carboxyl and 3-hydroxyl groups of UA affected UA potency, indicating that the interaction between pentacyclic triterpenoids and USP7 has unique stereospecificity. By thermal shift assay, the same authors demonstrated that UA interacts with USP7 in MM cell lines.

UA dose-dependently inhibited the proliferation of the MM with an IC50 of 6.56 μmol/L, accompanied by reductions in USP7 substrates such as MDM2, UHRF1 and DNMT1. Overexpression of USP7 partially abrogated UA-induced cell death, as well as the downregulation of its substrates [86]. Therefore, although UA does not directly bind the proteasome subunits, it may induce anti-tumor activity in MM cells via inhibition of deubiquitinases.

**Table 2:** Name, 2D structure, natural source and biological effects of natural PIs in MM.

Name	2D Structure	Natural Source	Biological Effects	
			**In Vitro Studies**	**In Vivo Studies**
**Theaflavin**	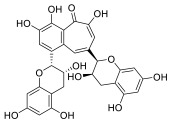	*Black tea*	increase in p53 reduction in NF-κB reduction in MAPK	n.a.
**Theaflavin-3-gallate**	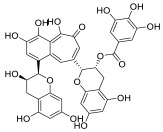			
**Theaflavin-3′-gallate**	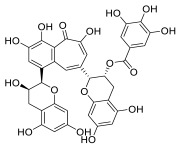			
**Theaflavin-3, 3′-digallate**	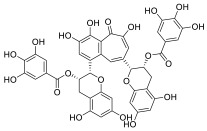			
**Hib-ester**	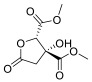	*Hibiscus sabdariffa*	n.a.	n.a.
**Hib-carbaldehyde**	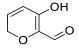			
**Celastrol**	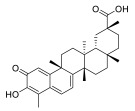	*Tripterygium wilfordii*	increase of apoptosis	decrease in the growth of MM cell line xenografts
**Syringolin**	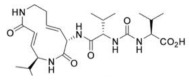	*Pseudomonas syringae pv. Syringae*	increase in p53	decrease in the growth of MM cell lines xenografts; reduced MM-induced bone lesions in mice
**TIR-199**	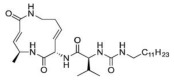	*–*		
**Tyropeptin A**	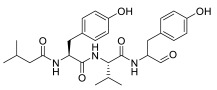	*Actinomycete strain MK993-dF2*	n.a.	
**AS-06**	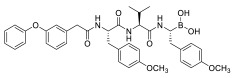	*-*	increase of apoptosis	decrease in the growth of MM cell lines xenografts
**AS-29**	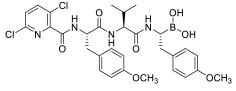	*-*		
**Pristimerin**	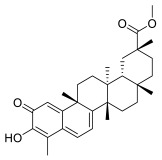	*Myatenus, Crossopetalum, Celastrus, Cheiloclinium, Mortonia*	reduction in NF-κB reduction in cyclin D	n.a.
**Ursolic acid**	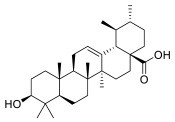	*Apple peel*	reduction of STAT3 reduction of MDM2 reduction of UHRF1 reduction of DNMT1	n.a.

## 4. Conclusions

Although PIs are the mainstay for the treatment of MM, the high risk of emergence of drug resistance constantly prompts the development of new drugs for the treatment of MM, especially in the relapsed and refractory setting.

In this regard, since ancient times natural products and their structural analogues have attracted scientific attention due to their great potential in the development of new drugs useful for various diseases, including cancer [87,88].

Natural products currently play a relevant role in cancer therapy, with a substantial number of anticancer agents used in the clinic being either natural or derived from natural products from various sources such as plants, animals or microorganisms from the marine environment. Accordingly, more than 50% all molecules approved between 1981 and 2014 are either natural products or their derivatives, with the majority acting as antitumor agents [17].

In this this review, we summarized the literature findings on the PI activity of natural compounds in MM, which began with the successful and exciting discovery of marizomib, a marine natural irreversible PI extracted from the marine actinomycete *Salinospora tropica*, under clinical development for the treatment of MM [89,90].

The case of marizomib underlines the pivotal role that natural compounds play in the anti-MM drug discovery process.

In fact, almost 100 natural compounds with documented anti-MM activity in vitro have recently been described [45], but roughly 10 have been found to act as PIs and were therefore discussed here. It is noteworthy that natural compounds such theaflavin, celastrol, syringolin, tyropepsin A and pristimerin can directly bind the β5 subunit, thus inhibiting the 26S proteasome and triggering anti-tumor activity. 

Overall, these compounds have been studied in vitro in MM cell lines and/or in subcutaneous xenografts as in vivo models; to clearly address their anti-MM potential, however, it is mandatory to study their activity in systems recapitulating the tumor microenvironment, which overall protects MM cells and induces drug resistance. In addition, it is necessary to further investigate, using adequate in vitro and in vivo models, whether the aforementioned natural compounds lack the common side effects (cardiotoxicity, peripheral neuropathy) observed with current PIs. At the molecular level, the biological sequelae underpinning the anti-MM activity of these compounds points to a significant and well-tolerated anti-tumor activity, which needs to be further dissected by performing additional mechanistic studies. Finally, it also appears to be mandatory to perform combination studies using natural PIs together with other anti-MM drugs.

In conclusion, the studies reported in this review underline the potential of natural PIs and, considering the enormous potential of these products, they could likely represent the starting point for *hit* identification and/or for the optimization of new and most active derivatives with stronger potency.

## Data Availability

Not applicable.
